# Monophasic Pulsed 200-μA Current Promotes Galvanotaxis With Polarization of Actin Filament and Integrin α2β1 in Human Dermal Fibroblasts

**Published:** 2016-01-19

**Authors:** Mikiko Uemura, Noriaki Maeshige, Yuka Koga, Michiko Ishikawa-Aoyama, Makoto Miyoshi, Masaharu Sugimoto, Hiroto Terashi, Makoto Usami

**Affiliations:** ^a^Department of Biophysics, Kobe University Graduate School of Health Sciences, Kobe, Hyogo, Japan; ^b^Department of Rehabilitation, Yoshida Hospital, Kobe, Hyogo, Japan; ^c^Department of Rehabilitation Science, Kobe University Graduate School of Health Sciences, Kobe, Hyogo, Japan; ^d^Faculty of Rehabilitation, Kobe Gakuin University, Kobe, Hyogo, Japan; ^e^Department of Plastic and Reconstructive Surgery, Kobe University Graduate School of Medicine, Kobe, Hyogo, Japan

**Keywords:** electrical stimulation, fibroblast, galvanotaxis, integrin α2β1, lamellipodia

## Abstract

**Objective:** The monophasic pulsed microcurrent is used to promote wound healing, and galvanotaxis regulation has been reported as one of the active mechanisms in the promotion of tissue repair with monophasic pulsed microcurrent. However, the optimum monophasic pulsed microcurrent parameters and intracellular changes caused by the monophasic pulsed microcurrent have not been elucidated in human dermal fibroblasts. The purpose of this study was to investigate the optimum intensity for promoting galvanotaxis and the effects of electrical stimulation on integrin α2β1 and actin filaments in human dermal fibroblasts. **Methods:** Human dermal fibroblasts were treated with the monophasic pulsed microcurrent of 0, 100, 200, or 300 μA for 8 hours, and cell migration and cell viability were measured 24 hours after starting monophasic pulsed microcurrent stimulation. Polarization of integrin α2β1 and lamellipodia formation were detected by immunofluorescent staining 10 minutes after starting monophasic pulsed microcurrent stimulation. **Results:** The migration toward the cathode was significantly higher in the cells treated with the 200-μA monophasic pulsed microcurrent than in the controls (*P* < .01) without any change in cell viability; treatment with 300-μA monophasic pulsed microcurrent did not alter the migration ratio. The electrostimulus of 200 μA also promoted integrin α2β1 polarization and lamellipodia formation at the cathode edge (*P* < .05). **Conclusion:** The results show that 200 μA is an effective monophasic pulsed microcurrent intensity to promote migration toward the cathode, and this intensity could regulate polarization of migration-related intracellular factors in human dermal fibroblasts.

Electrical stimulation is recommended for treating pressure ulcers (PUs) in Prevention and Treatment of Pressure Ulcers: Clinical Practice Guideline edited by the National Pressure Ulcer Advisory Panel, European Pressure Ulcer Advisory Panel, and Pan Pacific Pressure Injury Alliance in 2014,^1^ and monophasic pulsed microcurrent (MPMC) stimulation is one of the effective electrical therapies. Several clinical studies^2^^-^4 have reported that MPMC stimulation shortened the healing period for PUs; however, the parameters of MPMC, including intensity and polarity, are not identical in each clinical study.

Migration of fibroblasts toward the wound site is important to promote the formation of granulation tissue,^5^ and galvanotaxis is a crucial factor to promote migration. Our previous study^6^ showed that migration toward the cathode was greater in human dermal fibroblasts (HDFs) treated with 100-μA MPMC than in controls treated with 0-μA MPMC. Furthermore, PU healing was promoted by MPMC of 50 to 100 μA, with the cathode contacting the wound site in our clinical studies.^7^^,^^8^ These studies suggested that cathodal microcurrent intensity promotes galvanotaxis of fibroblasts toward the cathode, as well as wound healing. However, the intracellular change induced by MPMC stimulation and the effects of current intensities greater than 100 μA are not clear.

Integrin is a cell surface adhesion protein that activates outside-in signaling, leading to regulation of several cellular functions including cytoskeletal dynamics.^9^ In fibroblasts, integrin α2β1 binds to collagen and mediates migration and fibrosis.^10^ Integrin-triggered signaling induces actin polymerization such as lamellipodia at the leading edge.^11^ When cells migrate, lamellipodia formation occurs on the leading edge and initiates migration.^12^ Therefore, regulation of lamellipodia formation can affect cell migration. However, no study has investigated the effect of MPMC on lamellipodia formation in HDFs.

We hypothesized that HDFs migrate toward the cathode by MPMC stimulation and that optimum intensity for migration could alter migration-related intracellular factors such as integrin α2β1 and lamellipodia. For effective and safe treatments, it is important to elucidate the influence of MPMC on cell dynamics and intracellular alteration. Therefore, we examined the optimum intensity of MPMC for cell migration and the effects of MPMC on integrin α2β1 polarization and lamellipodia formation in HDFs.

## METHODS

### Cell culture

HDFs (CC-2511; Clonetics, San Diego, Calif) were grown in Dulbecco's Modified Eagle's Medium (Wako, Osaka, Japan) supplemented with 10% fetal bovine serum (Nichirei, Tokyo, Japan) in 100-mm tissue culture dishes (Iwaki, Tokyo, Japan) in a CO_2_ incubator at 37°C. Fibroblasts that had undergone 3 to 7 passages were used for the experiments.

### Cell migration assay

**Figure 1:**
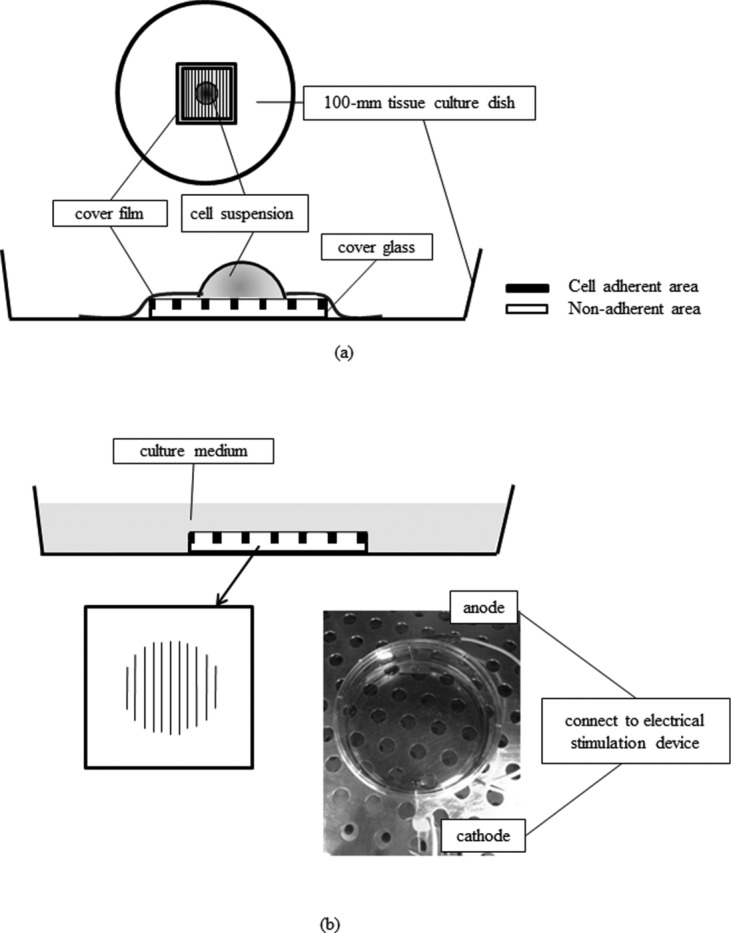
Schema of procedure of cell seeding and MPMC stimulation: (a) Cell seeding; (b) MPMC stimulation. MPMC indicates monophasic pulsed microcurrent.

Cover glasses (Cytograph L60S300; Dai Nippon Printing, Tokyo, Japan) were attached to the center of 100-mm tissue culture dishes and covered with a film that had a 14-mm hole in the center. The cover glasses contained 60-μm wide grooves for cell attachment that were divided by a 300-μm wide non–cell-adherent area, which was the best width for observing fibroblast migration in the preliminary study. For the experiments, fibroblasts (1.63 × 10^4^ cells) were seeded on the cover glass in a circular pattern through the hole at the center of the cover film and cultured for 24 hours (Fig 1*a*) according to the manufacturer's instruction. To identify the baseline for measuring the migration distance, the baseline was marked with a point on the bottom of the dishes. After incubation for 24 hours, the cover film was removed, the dish was filled with culture medium, and the electrical stimulation experiment was conducted with a dedicated electrical stimulation device (Fig 1*b*). The electrical stimulation procedure was performed as in our previous study.^6^ Platinum electrodes (20 × 5-mm plates) were used to prevent metal ion toxicity. MPMC (frequency, 0.3 Hz; pulse duration, 250 milliseconds) stimulation of the fibroblasts was conducted in a CO_2_ incubator at 37°C for 8 hours with current intensities of 0 (control), 100, 200, and 300 μA. Since reverse current could be generated after monophasic pulsed current stimulation,^6^ we connected the anode and the cathode with electrical ground cable for 1 minute after the MPMC stimulation to prevent this potential side effect in the present study. To analyze cell migration induced by MPMC, the cells were observed with a microscope under 50× magnification (Axiovert 25; Carl Zeiss, Oberkochen, Germany). Images were taken before MPMC stimulation and 24 hours afterward with a digital camera (Camedia c-5050 zoom; Olympus, Tokyo, Japan), and the photographs were synchronized with computer software (e-Tiling). A baseline perpendicular to the grooves was drawn from the initial point placed on the back of the dishes. For each group, we analyzed 10 grooves in the center of the cell attachment area and measured the distance of cell attachment area from the baseline toward the anode or cathode. To assess the migration, we calculated the distance between the cell attachment area before and after the MPMC stimulation to obtain the migration distance. The results were expressed as a migration rate, a ratio of the migration distance toward the cathode versus towards the anode.

### Trypan blue exclusion test

We used a trypan blue exclusion assay to assess the cell number and viability following the MPMC stimulation. The 25-mm cover glasses (Matsunami, Osaka, Japan) were placed in each well of a 6-well plate (Iwaki), and fibroblasts (28 × 10^4^ cells/well) were seeded into the well and cultured for 24 hours, after which we transferred the cover glass to the center of a 100-mm culture dish. The fibroblasts were electrostimulated for 8 hours in the incubator, and the assay was conducted at 8 hours after the start of electrical stimulation. The number of living and dead cells was counted with a hemocytometer under a light microscope.

### Immunofluorescence staining

Fibroblasts (5 × 10^4^ cells/well) were seeded into the 25-mm cover glasses placed in the well of a 6-well plate and cultured for 10 minutes. Next, the cells received MPMC stimulation for 2 hours in a CO_2_ incubator with an intensity of 0, 100, 200, or 300 μA. Fibroblasts were fixed with 4% paraformaldehyde and permeabilized with 0.2% Triton-X and then incubated with mouse anti-integrin α2β1 antibody, clone BHA2.1 (1:50; Merck Millipore, Darmstadt, Germany), overnight at 4°C. Cells were washed with phosphate-buffered saline and incubated with Alexa Fluor 594 goat anti-mouse IgG (H+L) secondary antibody (1:500; Life Technologies, Carlsbad, Calif) for 1 hour at room temperature. Following this staining, phalloidin-Alexa Fluor 488 (1:40; Life Technologies) was applied for 20 minutes and DAPI (1:1000; Dojindo, Kumamoto, Japan) for 3 minutes at room temperature. The coverslip was mounted with mounting medium (Fluorescent Mounting Medium; DAKO, Carpinteria, Calif). The stained cells were observed with a fluorescence microscope (Axiovert A1; Carl Zeiss).

### Integrin α2β1 polarization assay

The central 10 fields were selected in each sample. To quantify integrin α2β1 polarization, we divided each cell membrane in half, based on the side adjacent to the cathode or anode, and demarcated the area using an image tool (Image J). In each cell, the average fluorescence intensities for integrin α2β1 of cathode-adjacent (CA) and anode-adjacent (AA) cell membrane were corrected for whole average intensity. Asymmetry index was calculated and used to analyze the polarization according to the Finkelstein et al^13^ study. We calculated asymmetry index in each cell and the average of all the cells. Asymmetry index = (average of AA - average of CA)/average of whole cell.

### Lamellipodia formation

We observed lamellipodia formation in the same fields that were analyzed for asymmetry index. We classified F-actin–rich membrane extensions as lamellipodia according to the Steffen et al^14^ study. The ratio of lamellipodia formation at the cathode facing to that at the anode facing was calculated in each sample by counting the number of lamellipodia in each edge under the fluorescence microscope (100× magnification).

### Statistical analysis

The differences in the migration ratios, cell numbers, cell viabilities, asymmetry index, and lamellipodia formation ratio between the control and electrostimulus groups were analyzed with a Tukey-Kramer multiple-comparisons test. Differences with *P* < .05 were considered to be significant.

## RESULTS

### MPMC promotes galvanotaxis in HDFs

**Figure 2:**
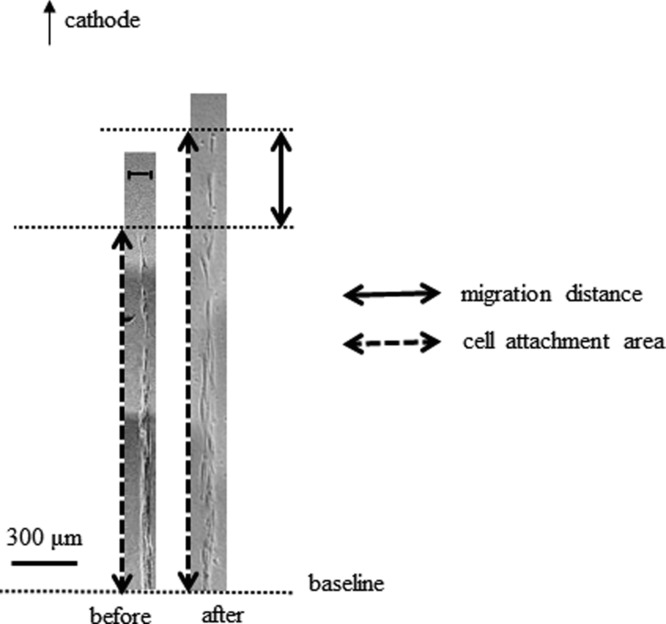
The migration distance in the 200-μA group. Representative migration of the groove was indicated by phase-contrast microscopy. Magnification: 400×. Scale bar: 300 μm. The distances of cell attachment area before and after MPMC stimulation were evaluated, and these differences were calculated. In the MPMC group, fibroblasts migrated toward the cathode. MPMC indicates monophasic pulsed microcurrent.

**Figure 3:**
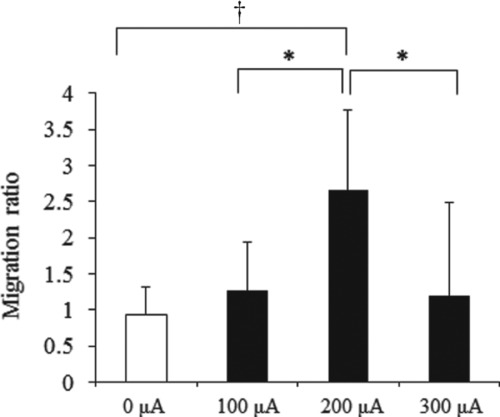
The ratio of migration length toward cathode to anode. The migration ratio for the 0-, 100-, 200-, and 300-μA groups were evaluated (*n* = 8–10). At 200 μA, fibroblast migration toward the cathode was significant (*P* < .01), but at 300 μA, the migration ratio decreased to less than the ratio at 100 μA. Data were expressed as mean ± SD. *P < .01 and †*P* < .05, Tukey-Kramer.

**Figure 4:**
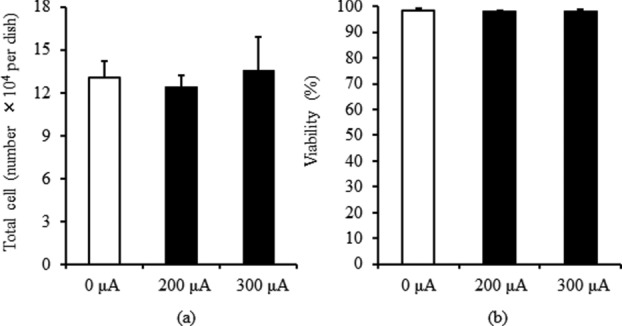
Cell number and cell viability by MPMC stimulation of more than 200 μA. Cell number (a) and cell viability (b) were measured by cell count using trypan blue staining (*n* = 6). There were no significant differences between the 0-μA and MPMC groups. Data were expressed as mean ± SD and analyzed by the Tukey-Kramer test. MPMC indicates monophasic pulsed microcurrent.

In MPMC groups, fibroblasts migrated toward the cathode (Fig 2). The migration ratio for the 0-, 100-, 200-, and 300-μA groups was 0.93 ± 0.13, 1.27 ± 0.25, 2.65 ± 0.37, and 1.19 ± 1.70, respectively (Fig 3). At 200 μA, fibroblast migration toward the cathode was significant (*P* < .01), but at 300 μA, the migration ratio decreased to less than the ratio at 100 μA. To investigate whether the high migration ratio in the 200-μA group and the decrease in the 300-μA group were caused by cell proliferation or cell toxicity by MPMC, we analyzed cell number and viability in the 200- and 300-μA groups using the trypan blue assay. There were no appreciable differences in the cell numbers or viability between the 0-μA and MPMC groups (Fig 4). Therefore, the increase in the migration ratio was caused by the MPMC promoting the migration of the fibroblasts.

### Integrin α2β1 polarization

**Figure 5:**
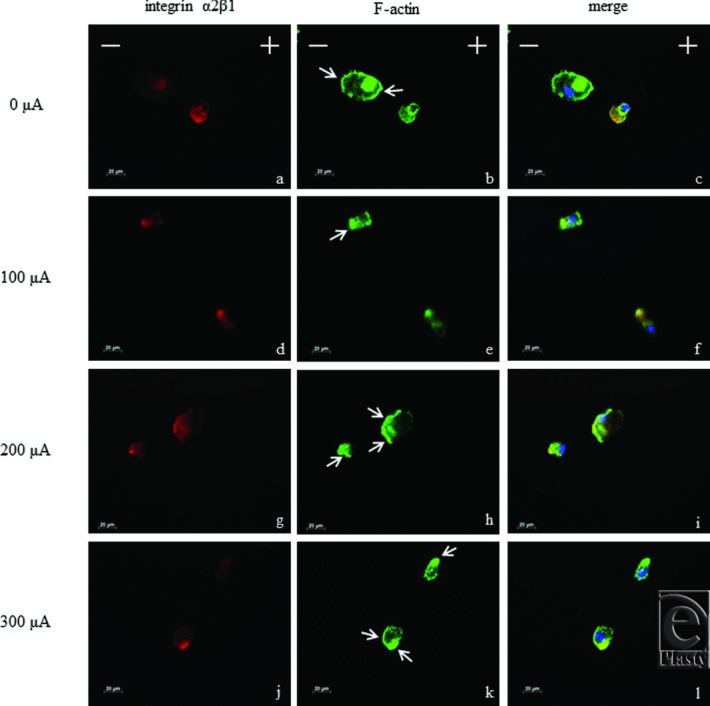
Integrin α2β1 polarization and lamellipodia formation in fibroblasts. Immunofluorescence images of integrin α2β1 (red), F-actin (green), and nuclei (blue) staining. Integrin α2β1 was observed over the entire cell surface in 0 μA (a), whereas integrin α2β1 polarized in cell edges in MPMC groups (d, g, j). Lamellipodia (arrows) were formed in the cathode and anode facing at 0 μA (b). In MPMC groups, lamellipodia formation was observed at cell edge of the cathode or anode (e, h, k). The images of integrin α2β1, F-actin, and nuclei were superimposed (c, f, i, l) with AxioVision software (Zeiss, Oberkochen, Germany). MPMC indicates monophasic pulsed microcurrent.

**Figure 6:**
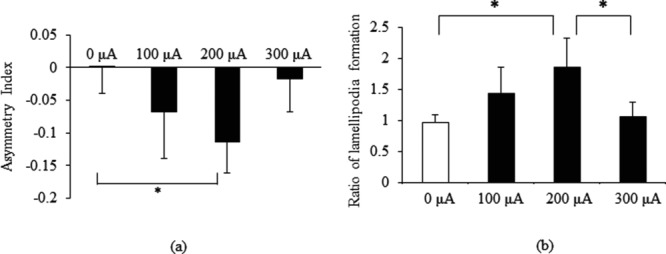
Asymmetry index of integrin α2β1 and ratio of lamellipodia formation at the cathode to anode edge. (a) Integrin α2β1 polarization was assessed by asymmetry index. Integrin α2β1 was significantly polarized to the cathode at 200 μA (*n* = 4). (b) The number of lamellipodia formation at the cathode edge and anode edge was counted and calculated as the ratio of lamellipodia formation at the cathode to anode. Lamellipodia formation at the cathode edge was significantly higher at 200 μA than at 0 and 300 μA (*n* = 4). Data were expressed as mean ± SD. *P < .05, Tukey-Kramer.

A negative asymmetry index indicates that integrin α2β1 polarizes to the cathode. In the 0-μA group, integrin α2β1 was observed over the entire cell surface, whereas with MPMC groups, integrin α2β1 polarized to the edge of the cell as shown in Figures 5*a*, *d*, *g*, and *j*. Figure 6*a* shows that integrin α2β1 polarization to the cathode is significantly higher in the 200-μA group than in the 0-μA group as determined by analysis of asymmetry index (*P* < .05). Similar to the result for migration, when stimulated with 300 μA, integrin α2β1 did not polarize to the cathode.

### Lamellipodia formation in electrical stimulation

We next investigated whether MPMC influences actin organization by staining for F-actin. Lamellipodia, the projections on the leading edge of the cell, propel cell migration. Figures 5*b* and *k* show that lamellipodia were formed at both the cathode and anode edges in the 0- and 300-μA groups, whereas in the 200-μA group, they were observed at the cathode edge (Fig 5*h*). The ratio of lamellipodia formation at the cathode edge versus the anode edge was significantly higher in the 200-μA group than in the 0- and 300-μA groups (Fig 6*b*; *P* < .05). Lamellipodia polarization stimulated by 200 μA indicates that MPMC at this intensity can directly affect the assembly of actin filaments.

## DISCUSSION

We found that MPMC of 200 μA promoted galvanotaxis of HDFs toward the cathode. Moreover, it appeared that the integrin α2β1 polarization and lamellipodia formation observed at the cathode edge are related to μA intensity up to 200 μA. These findings suggest that the MPMC of optimum intensity and polarity is important to promote migration of fibroblasts, and the effective intensity in migration could regulate the polarization of migration-related intracellular factors.

MPMC of 200 μA strongly increased migration ratio toward the cathode; however, stimulation of 300 μA demonstrated a lower migration ratio than 200 μA and a ratio similar to the 100-μA group. Although some in vitro studies^15^^-^18 also showed that fibroblasts migrated toward the cathode by electrical stimulation, these studies did not assess the dependency on intensity in the migration. The present study revealed an optimum microcurrent intensity of 200 μA to promote galvanotaxis of HDFs with the single-peak change of migration. This single peak observed in HDFs is in agreement with the study using neutrophils showing that migration distance was inhibited at intensities greater than the optimum intensity of 60 μA.^19^ These results suggest that each cell type has an optimum intensity for galvanotaxis.

With an MPMC of 200 μA, polarization of integrin α2β1 and lamellipodia formation were observed at the cathode edge. Our results for integrin polarization by MPMC agree with those of ligament fibroblasts assessed by difference in volts.^15^ Meanwhile, at amplitudes of 100 and 300 μA, polarization of integrin α2β1 and lamellipodia formation toward the cathode were not observed. The present study is the first demonstration of these single-peak changes in migration-related intracellular factors in HDFs by MPMC stimulation. Moreover, the migration assay and immunofluorescence analysis found consistency in the optimum MPMC intensity between the promotion of migration and the polarization of migration-related intracellular factors. Actin polymerization at the leading edge is necessary for cell migration, and Li and Kolega^20^ demonstrated that an electric field induced direct migration of bovine vascular endothelial cells to the cathode and concentration of F-actin at the cathode. Although we could not analyze lamellipodia formation of the HDFs in the migration assay, the action of MPMC on lamellipodia polarization may promote cell migration. Focal adhesion kinase (FAK) is one of the scaffold proteins of integrin. Small electric fields were shown to activate FAK,^21^ and integrin-activated FAK leads to lamellipodia formation and cell migration.^22^ These results suggest that MPMC polarized integrin α2β1 at the cathode edge and then outside-in signaling occurred, leading to actin polymerization. Therefore, integrin α2β1 may play a role as a mechanosensor of MPMC. However, the relationship between migration and both integrin α2β1 polarization and lamellipodia formation is still incompletely understood in the present study. Further studies are needed to investigate other factors of outside-in signaling, including FAK.

This study revealed that MPMC of 200 μA influenced in vitro galvanotaxis of fibroblasts toward the cathode in accordance with distribution of integrin α2β1 and actin filaments. Indeed, in vivo research has shown that measurable microcurrent generated following mammalian skin injury accompanies tissue healing.^23^ Therefore, in clinical trials, it is important to determine the current intensity, polarity, and state of target tissues for healing PUs and other chronic wounds. On the basis of the results of the present study, future investigations should be conducted to determine the effective parameters on the frequency and treatment time, as well as the influence on integrin trigger-related signaling.
